# Erythrocyte transketolase activity coefficient (ETKAC) assay protocol for the assessment of thiamine status

**DOI:** 10.1111/nyas.14547

**Published:** 2020-12-22

**Authors:** Kerry S. Jones, Damon A. Parkington, Lorna J. Cox, Albert Koulman

**Affiliations:** ^1^ NIHR BRC Nutritional Biomarker Laboratory, MRC Epidemiology Unit University of Cambridge Cambridge UK; ^2^ MRC Elsie Widdowson Laboratory Cambridge UK

**Keywords:** thiamine, vitamin B1, ETKAC, beriberi

## Abstract

Vitamin B1 (thiamine) is an essential nutrient that acts as a cofactor for a number of metabolic processes, particularly in energy metabolism. Symptoms of classic thiamine deficiency are recognized as beriberi, although clinical symptoms are nonspecific and recognition of subclinical deficiency is difficult. Therefore, reliable biomarkers of thiamine status are required. Thiamine diphosphate is a cofactor for transketolase, including erythrocyte transketolase (ETK). The ETK activity assay as an indirect, functional marker of thiamine status has been used for over 50 years. The ETK activity assay provides a sensitive and specific biomarker of thiamine status; however, there is a lack of consensus over the cutoffs for deficiency, partly due to a lack of assay harmonization. Here, we provide a step‐by‐step protocol for the measurement of ETK activity and the calculation of the ETK activity coefficient, including detailed explanations of equipment and chemicals required and guidance for quality control procedures. Harmonization of the protocol will provide the basis for the development of internationally recognized cutoffs for thiamine insufficiency. The establishment of quality control materials and a quality assurance scheme are recommended to provide reliability. This will ensure that the ETK activity assay remains an important method for the assessment of thiamine status.

## Graphical abstract

Reliable biomarkers of thiamine status are required to help diagnose thiamine deficiency. Thiamine diphosphate (ThDP) is a cofactor for transketolase, including erythrocyte transketolase (ETK). The ETK activity assay provides a sensitive and specific biomarker of thiamine status; however, there is a lack of consensus over the cutoffs for thiamine deficiency, partly due to a lack of assay harmonization. Here, we provide a step‐by‐step protocol for the measurement of ETK activity and the calculation of the ETK activity coefficient (ETKAC).

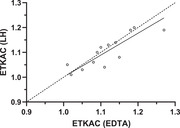

## Introduction

Thiamine (vitamin B1) is an essential micronutrient. In humans, the majority of thiamine exists in phosphorylated forms: thiamine monophosphate, thiamine diphosphate (ThDP) (also known as thiamine pyrophosphate), and thiamine triphosphate. ThDP is the major form, making up around 80% of total blood thiamine and is mainly present in erythrocytes and white blood cells.

ThDP is an essential cofactor for a number of metabolic enzymes, particularly those associated with oxidative and nonoxidative carbohydrate metabolism. These include pyruvate dehydrogenase (responsible for the conversion of pyruvate to acetyl‐coenzyme A), α‐ketoglutarate dehydrogenase in the Krebs cycle, and the branched chain α‐keto acid dehydrogenase complex. In nonoxidative carbohydrate metabolism, ThDP is a cofactor for the transketolase in the pentose phosphate pathway, necessary for the production of pentoses and NADPH used in nucleic acid and fatty acid synthesis, respectively.^1^

Thiamine deficiency is associated with nonspecific clinical symptoms affecting the cardiovascular, muscular, and nervous systems. The classical manifestation of thiamine deficiency is beriberi. The populations most at risk are breastfed infants of thiamine‐deficient mothers in low‐income countries, particularly those with poor diet diversity and where rice is the staple food.^2^, ^3^ In high‐income countries, deficiency is rare but may be present in the elderly, chronic alcoholics, and persons with acute or chronic medical conditions associated with malnutrition.^1^ In addition to the effect of acute deficiency, evidence also exists that subclinical deficiency may have long‐term effects on cognition and gross motor skills.^2^

In order to investigate the potential impact of subclinical deficiency and to improve our understanding of the global prevalence of thiamine deficiency, biomarkers to assess thiamine status are essential. The measurement of serum or plasma thiamine concentration is of limited use since it represents only a small portion of thiamine in the blood and is affected by a number of disease states.^1^ Although a correlation between plasma thiamine and erythrocyte ThDP has been shown,^4^ these data were from a thiamine fortification trial; plasma thiamine is affected by recent dietary intake and is, therefore, not suitable as a biomarker of long‐term thiamine status. Urinary thiamine excretion may correlate with all levels of thiamine intake but low intakes. However, it does not provide information on thiamine status, particularly when status is low, as thiamine may be conserved. In addition, single 24‐h urine collections are of limited use due to large within‐subject variability.^5^ Longer‐term thiamine status from human blood samples can be assessed either by direct measurement of ThDP in erythrocytes or whole blood, or by the measurement of the degree of ThDP‐saturation of erythrocyte transketolase (ETK), a ThDP‐dependent enzyme. The advantages and disadvantages of both the direct and indirect assays were reviewed recently,^1^, ^2^, ^6^ including a detailed description of the direct measurement of ThDP. However, the methodology to measure ETK activity has received less attention.

ETK activity is commonly expressed as a ratio or “activity coefficient” (ETKAC). Two measurements are made to determine the EKTAC: (1) the basal activity of ETK and (2) “activated” or “stimulated” ETK activity by the addition of exogenous excess ThDP. The ratio of activated to basal activity gives the ETKAC and provides a proxy measure for the *in vivo* activity of ETK and an indication of the availability of ThDP and thiamine status.

In this manuscript, we describe the background to the ETK activation assay and discuss aspects relating to reliability and robustly performing, reporting, and interpreting the ETK activity data. In addition, we present a step‐by‐step protocol (see File S1, online only) that provides detailed guidance and should enable laboratories to set up the method independently and improve harmonization.

## Historical background

The ETK activity assay in its early form for the application of thiamine deficiency assessment was established by Brin^7^ and was later improved and adapted as understanding and technology progressed. Assays moved from the less specific colorimetric determination of substrate disappearance or appearance of end products to UV detection of NADH.^8^ The assay relies on the detection of small changes in enzyme activity and early methods lacked the necessary sensitivity and had relatively poor precision. The assay was further refined and adapted for semiautomated clinical chemistry analyzers. This improved measurement precision, even at observed high absorbance, and the ability to run basal and activated measurements side by side further improved performance.^9^, ^10^ For additional convenience and to remove the need for large, expensive equipment, the assay has been further adapted to a 96‐well plate format and measurement with a plate reader.^11^ The assay as it is currently performed in our laboratory is described herein.

## Chemistry of the assay

The ThDP‐dependent enzyme ETK catalyzes the metabolism of pentoses in two reactions in the pentose phosphate pathway:

(1) xylulose‐5‐phosphate + ribose‐5‐phosphate ⇆ sedoheptulose‐7‐phosphate + glyceraldehyde‐3‐phosphate
(2) xylulose‐5‐phosphate + erthyose‐4‐phosphate ⇆ fructose‐6‐phosphate + glyceraldehyde‐3‐phosphate

**Figure 1:**
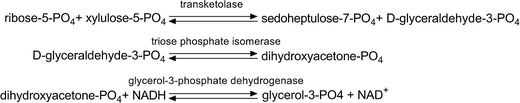
The enzymatic reactions in the ETK activity assay. The assay monitors the decrease in absorbance at 340 nm related to NADH oxidation.

In the current assay, the resultant glyceraldehyde‐3‐phosphate is metabolized to dihydroxyacetone phosphate, which in the final reaction is reduced to glycerol‐3‐phosphate by glycerol‐3‐phosphate dehydrogenase and NADH (Fig. 1). The assay monitors the rate of oxidation of NADH by measuring the decrease in absorbance at 340 nanometers. In the described protocol, xylulose‐5‐phosphate is not required as a substrate; ribose‐5‐phosphate is converted to xylulose‐5‐phosphate by the erythrocyte thiamine‐independent enzymes ribose phosphate isomerase and ribulose phosphate‐3‐epimerase.^8^

## Calculation and interpretation of the ETKAC

Calculation of the ETKAC in erythrocyte lysates involves: (1) measurement of the basal activity of ETK; and (2) measurement of activated or stimulated ETK activity by the addition of exogenous excess ThDP. The basal test represents the active holo‐transketolase activity. Addition of exogenous ThDP activates apo‐transketolase and the stimulated activity represents the activity of both the (now‐activated) apo‐transketolase and holo‐transketolase. Takeuchi *et al*. demonstrated in experiments with apo‐transketolase enzymes that the ETKAC reflects the degree of saturation of ETK with ThDP.^12^

The results of the basal and stimulated activities are commonly presented as a ratio or activity coefficient, the ETKAC calculated as (stimulated activity)/(basal activity). Alternatively, results may be presented as a percentage (% activation = (ETKAC × 100) – 100; also known as the thiamine pyrophosphate effect (TPPE)).

In thiamine sufficiency, addition of exogenous ThDP will make little difference to the enzyme activity and hence an ETKAC close to 1 will be obtained. In thiamine insufficiency or deficiency, the addition of exogenous ThDP has a progressively greater effect on ETKAC, which provides a continuum of thiamine status.^13^

Although there is no international consensus on cutoffs, the commonly used threshold for risk of deficiency is an ETKAC of >1.25.^13^, ^14^ An ETKAC of <1.15 indicates sufficiency and values between 1.15 and 1.25 suggest a low risk of clinical deficiency.^13^ Others have suggested values of ≥1.2 indicate deficiency.^15^ Beriberi is typically associated with ETKAC values >1.4.^2^

The use of ETKAC rather than absolute measures of ETK activity is preferred for three main reasons: (1) the between‐subject variation in basal activity is large; (2) it is assumed, but not certain, that apoenzyme levels are not affected by vitamin deficiencies;^5^ and (3) it reduces the need for the precise definition of assay conditions (e.g., optical path length), which are critical to calculate absolute enzyme activities.^16^ Others have suggested using combinations of the stimulated ETK activity and the ETKAC in interpretation.^17^

However, the use of basal ETK activity rather than ETKAC has also been recommended, particularly for the assessment of thiamine status in infants due to potential low levels of apoenzyme caused by long‐term low exposure to thiamine.^18^ A recent study of children and adults found that while both basal and ETKAC correlated with erythrocyte ThDP, the correlation was stronger for basal activity.^19^ Basal (or activated ETK) activity needs to be expressed per mass unit of hemoglobin, and it is necessary to measure the hemoglobin concentration in the same lysate as used for the ETK activity assay. In addition, it is necessary to calculate the factor needed to convert rate to activity.

## Recommended sample type

Washed erythrocytes are the recommended sample type. Leukocytes have relatively large amounts of thiamine, and transketolase activity can, therefore, be considerably higher in whole blood compared with erythrocytes.^8^, ^20^ The use of leukocytes^21^, ^22^ to assess transketolase activity has been reported but has not been applied routinely, possibly because the shorter half‐life of leukocytes compared with erythrocytes would result in a biomarker of thiamine status that is only short term.

The use of whole blood, a more convenient sample compared with washed erythrocytes, has also been considered. While transketolase activity was higher in whole blood, the transketolase activity coefficient was similar between whole blood and erythrocyte hemolysates.^20^, ^23^ However, there are concerns around the use of whole blood, partly due to between‐person variability in leukocyte count, particularly in clinical conditions that may impact leukocyte number.^24^

**Figure 2:**
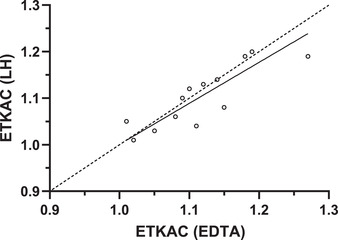
Deming regression of ETKAC measured in erythrocyte hemolysates collected in either ETDA or lithium heparin (LH) blood tubes from 15 adults. Open circles, observed data points; solid line, regression line; dashed line, line of equality. Slope (95% CI) = 0.88 (0.37–1.39); intercept (95% CI) = 0.26 (−0.44 to 0.68).

Whole blood collected in blood tubes containing either lithium heparin (LH) or ethylenediaminetetraacetic acid (EDTA) anticoagulant can be used as the starting sample for the preparation of washed erythrocytes. Agreement in our own laboratory between the two sample types is shown in Figure 2.

## Sample stability

A small number of studies have investigated the stability of ETK activity and ETKAC across a number of analytical stages, including in whole blood and hemolysates, and at a range of temperatures from refrigeration at 4 °C to frozen storage at −20 and −70 °C, possibly reflecting the availability of cold storage in different clinical and field site settings.

### Whole blood

In whole blood kept at 4 °C or at room temperature, the ETKAC was stable for up to 24 h, decreasing thereafter;^25^, ^26^ however, basal activity was reportedly stable for up to 4 days.^25^

**Figure 3:**
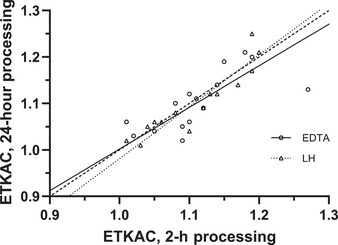
Deming regression of ETKAC measured in erythrocyte hemolysates prepared from LH and EDTA whole blood collected from 15 adults and processed within 2 h of collection or after refrigeration for 24 hours. Dashed line is the line of equality.

In our laboratory, whole blood samples from 15 participants kept at 4 °C were also observed to be stable for 24 h, with no significant change in the ETKAC observed in LH‐treated whole blood (% geometric mean change (95% confidence interval): −0.3 (−2 to 2)) or in EDTA‐treated whole blood (−1 (−3 to 1)), and they showed good agreement (Fig. 3).

### Washed erythrocytes

Puxty *et al*. observed greater deterioration of the ETKAC after storage of washed erythrocytes at −20 °C in participants in an insufficient group (ETKAC > 1.15) group than in a sufficient group (ETKAC < 1.15), and they speculate this may be due to the stability of different isoenzymes. In the sufficient group, samples were stable for up to 14 days at −20 °C but for only 4 days in the insufficient group.^26^ Washed erythrocytes stored at 4 °C were stable for less than 2 days.^26^ Other studies observed no consistent change over time in basal and stimulated activity.^9^

### Hemolysates

**Figure 4:**
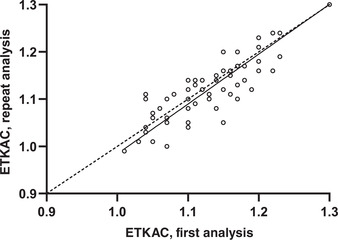
Deming regression of repeat analysis of erythrocyte hemolysates after storage for 6 months at −70 °C. Open circles, observed data points; solid line, regression line; dashed line, line of equality. Slope (95% CI) = 1.06 (0.92–1.20); intercept (95% CI) = −0.07 (−0.24 to 0.09) (*n* = 59).

Hemolysates can be kept frozen at −20 °C for at least 4 weeks; however, caution should be paid to the number of freeze‐thaw cycles since reports suggest that significant decreases in enzyme activity can occur after two freeze‐thaw cycles.^8^ Another study showed a decrease of 7% in the ETKAC in hemolysates analyzed after freezing at −18 °C for 2 weeks compared with fresh samples.^27^ As with washed erythrocytes, samples kept at −70 °C remained stable for at least 2 months.^25^ In our own laboratory, we observed a less than 1% change in the ETKAC in a set of 59 hemolysates stored at −70 °C for 6 months and excellent agreement across a range of EKTACs (Fig. 4). Enzyme activities reportedly remain stable at −70 °C for over 1 year.^2^ Storage of washed erythrocytes and hemolysates at −70 °C or below is recommended.^16^

## Assay and individual variability

### Analytical variability

Our routine method includes three internal QC materials with ETKAC (mean (SD)) of 1.07 (0.03), 1.15 (0.05), and 1.18 (0.05) and corresponding between‐batch %CVs of 3.1, 3.9, and 4.1% (*n* = 25). Targets of <5% are recommended.

### Within‐individual variation

A study from 1990 reported within‐individual variation of basal and activated transketolase activity between 3% and 6% measured in 20 samples collected from each of four individuals over 60 days on a constant diet.^28^

### Between‐individual

ETKAC data from the UK National Diet and Nutrition Survey (NDNS) Rolling Programme (Years 7–8 (2014–2016))^29^ are presented in Table 1 and show the range of values in a largely thiamine‐replete population. Between‐individual CV is around 5%. The 2.5th–97.5th percentile range in the UK population based on the NDNS Rolling Programme data for Years 1–8, using the ETKAC method described herein, is 1.01–1.21.

**Table 1:** ETKAC in UK population estimated from the UK National Diet and Nutrition Survey Rolling Programme, Years 7 and 8 (2014−2016)

			Risk of deficiency, %*^a^*		
	Mean ratio (SD)	*n*	Low EKTAC (<1.15)	Moderate EKTAC (1.15−1.25)	High EKTAC (>1.25)
Children 4−10 years old	1.10 (0.052)	101	80	20	0
Children 11−18 years old	1.12 (0.052)	164	68	32	0
Adults 19−64 years old	1.11 (0.052)	513	73	26	1
Adults 65+ years old	1.10 (0.062)	153	80	19	1

*^a^*Percentages are weighted to provide risk of deficiency estimates for the UK population.

## Limitations and other factors

Genetic differences leading to different transketolase isoforms that may have different activities or stabilities^30^ or be associated with the manifestation of thiamine deficiency in Wernicke–Korsakoff syndrome have been suggested.^30^, ^31^ However, other studies have found no evidence for isoforms or disease associations and suggest that earlier observations were due to the methodologies used.^32^, ^33^, ^34^, ^35^, ^36^

A venous whole blood sample is required for the determination of ETKAC (and whole blood ThDP). While a venous blood sample is a common sample type, collection and processing of blood samples in the field or limited‐resource settings present a number of challenges, including laboratory infrastructure for washing of erythrocytes and cold storage. Capillary blood sampling and dried blood spots (DBSs) provide a more convenient sample. Recently, Huang *et al*. described a DBS method for ThDP that showed a high degree of correlation with whole blood results (*r* = 0.964; *P* < 0.0001), although ThDP in DBSs was not stable at room temperature.^37^ Further work is required to determine the feasibility of measuring ETKAC in whole blood as either a venous sample or a DBS.

The impact of clinically observed thiamine deficiency, ThDP concentration, their effect on transketolase enzyme, and consequences for interpretation of transketolase activity have been a topic of discussion for many years.^17^, ^38^ It has been suggested that liver disease may directly affect apo‐transketolase concentration, causing apparently normal ETKAC in the presence of clinical symptoms of thiamine deficiency. However, other studies posit that the decrease in apo‐transketolase is a response to thiamine deficiency.^17^, ^38^ Either way, chronic thiamine deficiency may cause loss of apo‐transketolase and evidence suggests that this is caused by an effect on synthesis rather than catabolism of transketolase.^39^ The impact of this effect on interpretation of the EKTAC is, however, uncertain. Price *et al*. observed a lower activation after *in vitro* stimulation of ETK compared with *in vivo* activity following thiamine supplementation that may be related to irreversible inactivation of the enzyme *in vitro*.^40^ Thus, observations *in vitro* may not represent the *in vivo* situation. Clinical conditions, for example, certain anemias or diseases that affect erythrocyte cell survival time and the age distribution, may also affect the interpretation of ETKAC. Holo‐transketolase enzyme concentration is lower in older than younger erythrocytes^41^ and as holo‐transketolase decreases, there is a consequent increase in the ETKAC.^26^, ^41^

## Conclusions

The ETKAC assay provides an indirect, functional measure of thiamine status. Perceived limitations of the assay include relatively poor precision, lack of standardization, instability of the transketolase enzyme, and lack of consensus about cutoffs for deficiency.^42^ However, these limitations equally apply to other assays of thiamine status, such as whole blood ThDP. ETKAC has advantages over methods for the direct measurement of ThDP, including relative ease and the requirement for less specialized equipment, making the assay potentially more affordable and sustainable. Furthermore, ETKAC provides a potentially better marker of long‐term thiamine status that is less affected than ThDP by acute changes in thiamine intake.^43^

In this paper, we provide a detailed protocol for the measurement of ETK activity that can be used as a foundation for assay harmonization. There is also a need to develop an external quality assurance scheme to provide for the independent monitoring and assessment of laboratory performance. The establishment of a network of international laboratories willing to share samples for an interlaboratory comparison could provide a first step in the development of an external quality assurance scheme. The difficulty of obtaining blood samples in sufficient volume from thiamine‐deficient individuals results in a lack of quality control materials that include the thiamine‐deficient range. Therefore, there is a need to develop quality control and reference materials with consensus values spanning both sufficiency and deficiency, which can be supplied to laboratories in order to monitor, understand, and standardize assay performance.

Improvements in harmonization will also provide opportunity for investigating outstanding questions related to thiamine physiology and EKTAC assays, including the establishment of cutoffs for deficiency, characterizing agreement with other markers of thiamine status (e.g., ThDP), and to better understand conditions where interpretation of ETKAC may be confounded.

## Author contributions

K.S.J. and D.A.P. wrote the manuscript. L.J.C. designed the assay. L.J.C. and D.A.P. performed the experiments. K.S.J., D.A.P., L.J.C., and A.K. reviewed and approved the manuscript and accept responsibility for the contents.

## Competing interests

The authors declare no competing interests.

## Supporting information

**File S1**. Protocols for (1) processing of whole blood samples required to produce washed erythrocyte specimens for the erythrocyte transketolase activity coefficient (ETKAC) assay, and (2) the measurement of the erythrocyte transketolase activity coefficient (ETKAC).
**Table P1**. Reagents required for the ETKAC assay.
**Figure P1**. Plate map for the ETKAC analysis.
