# Supplementary material for: Erythrocyte transketolase activity coefficient (ETKAC) assay protocol for the assessment of thiamine status
Source: Ann N Y Acad Sci. 2020 Dec 22;1498(1):77–84. doi: 10.1111/nyas.14547 (PMC8451777; doi:10.1111/nyas.14547)
Supplement: Supplementary file 1 — File S1. Protocols for (1) processing of whole blood samples required to produce washed erythrocyte specimens for the erythrocyte transketolase activity coefficient (ETKAC) assay, and (2) the measurement of the erythrocyte transketolase activity coefficient (ETKAC). Table P1. Reagents required for the ETKAC assay. Figure P1. Plate map for the ETKAC analysis. [file NYAS-1498-77-s001.pdf]

ETKAC assay protocol
NIHR BRC Nutritional Biomarker Laboratory, University of Cambridge
Kerry S. Jones, Damon A. Parkington, Lorna J. Cox, Albert Koulman

## Supplementary Online Materials

## Protocol for processing of whole blood samples required to produce washed erythrocyte specimens for the erythrocyte transketolase activity coefficient (ETKAC) assay

### Materials

- Refrigerated centrifuge with shielded rotor (see **note 1**, below)
- Source of deionised water
- Saline. Approximately 30 ml of a 0.9% saline solution is required per participant for erythrocyte washing
- Pastettes
- Freezer (not self-defrosting) (see **note 2**, below)

### Sample processing procedure

1. Pre-chill centrifuge to 4 °C
2. Mix all blood containers by inverting slowly 10 times
3. Centrifuge the tubes at 4 °C according to manufacturer's instructions or local protocols for collection of plasma
4. Remove the plasma according to local protocols
5. Remove buffy coat top layer from the tube using a Pastette
6. Re-suspend the erythrocytes in normal saline (0.9% w/v NaCl solution) with approximately 2 times the volume of the erythrocytes. Gently invert the tube 10 times to mix
7. Centrifuge the tubes at 4 °C and 2000 × *g* for 10 minutes and discard the supernatant
8. Repeat steps 6 and 7 twice for a total of 3 washes or until the supernatant is clear
9. Completely remove the supernatant
10. Washed erythrocytes should be stored at −70 °C

------------------------------------------------------------------------------------------------------------

*Notes for processing whole blood samples.*
**Note 1.** If a refrigerated centrifuge is not available, then pre-cooling of centrifuge buckets and blocks at +4 °C is recommended.
**Note 2**. Washed erythrocyte samples should be frozen at −70 °C. Where this is not possible, samples should be stored at maximum -20 °C and transferred to −70 °C or below as soon as possible.

## Protocol for the measurement of erythrocyte transketolase activity coefficient (ETKAC)

### <u>Materials</u>

### Equipment

- Incubator cabinet set at 37 °C
- Thermo Multiskan FC plate reader with incubator and 340 nm filter (Thermo Fisher Scientific, UK. Catalogue Number: 51119100) (see **note 1**, below), programmed appropriately for this assay
- Single channel pipettes, suitable for delivering 50 µl and 30 µl with correct tips (see **note 2**, below)
- Multichannel pipettes suitable for delivering 15 µl, 200 µl and 500 µl with correct tips (see **note 2**, below)
- Refrigerated centrifuge with shielded rotor
- pH meter
- Orbital plate shaker with speed control
- Glassware: 500 ml & 1 l graduated glass bottles, 1 l glass measuring cylinder

ETKAC assay protocol
NIHR BRC Nutritional Biomarker Laboratory, University of Cambridge
Kerry S. Jones, Damon A. Parkington, Lorna J. Cox, Albert Koulman

**Consumables**

- 50 ml Falcon tubes (Corning Optical Communications, Flintshire, UK Catalogue Number: CLS430829), or equivalent
- 1 or 2 ml Pastettes
- Troughs for multichannel pipettes
- Greiner UVstar 96-well plates (Greiner Bio-One Ltd, Gloucestershire, UK. Catalogue Number: 655801), or equivalent (must be flat-bottomed and UV transparent at 340 nm)
- Screw-cap 2 ml Sarstedt tubes (Satstedt Ltd., Leicester, UK Catalogue Number: 72.730.406 ) or equivalent
- Self-adhesive plate sealers
- Source of deionised water (recommended resistivity of > 18.2 MΩ.cm)

**Method**

1. **Reagent preparation (Table P1)**

**1.1. 2M Hydrochloric Acid**

- Add 417 ml deionised water to a gradated glass container
- In a fume cupboard, make up to 500 ml with 37% hydrochloric acid
- Store at room temperature

**1.2. Tris buffer**

- Dissolve 12.1 g Trizma base in 700–800 ml deionised water. Buffer to pH 7.6 with 2M hydrochloric acid; make up to 1 l, mix by inversion
- Store at +4 °C for up to one month

**1.3. 10% Tween 20 (see note 3**, below**)**

- Measure 9 ml deionised water into a graduated tube
- Add 1 ml Tween 20 below the meniscus using a Pastette, using the water meniscus and graduations to determine the volume (see **note 4**, below)
- Cap the tube and mix by inversion
- Store at +4 °C for up to 6 months

**1.4. 1% Tween 20**

- Add 1 ml of 10% Tween 20 into a graduated tube using a pipette
- Make up to 10 ml with deionised water
- Cap the tube and mix by inversion
- Store at +4 °C for up to 6 months

**1.5. Thiamine pyrophosphate (TPP) (thiamine diphosphate, ThDP)**

- Prepare a solution of 3.6 mg/ml TPP in Tris buffer. 1 ml is needed per plate, approximately 0.2–0.5 ml pipetting excess recommended
- Weigh between 4.5 and 5.5 mg of TPP and calculate the volume of Tris buffer required; for example: 5 mg TPP / 1.39 ml Tris buffer
- Prepare on day of analysis
- Store at +4 °C

**1.6. Ribose-5-phosphate (R5P)**

- Prepare a solution of 82.5 mg/ml R5P in Tris buffer
  1 ml is needed per plate, approximately 0.2–0.5 ml pipetting excess recommended
- Weigh between 100 and 120 mg of R5P and calculate the volume of Tris buffer required; for example: 100 mg R5P / 1.21 ml Tris buffer
- Prepare on day of analysis
- Store at +4 °C

**1.7. Nicotinamide adenine dinucleotide (NADH)**

- Prepare a solution of 3.75 mg/ml NADH in Tris buffer
  1 ml is needed per plate, approximately 0.2–0.5 ml pipetting excess recommended
- Weigh between 4.5 and 6 mg of NADH and calculate the volume of Tris buffer required; for example: 5 mg NADH / 1.33 ml Tris buffer
- Prepare on day of analysis
- Store at +4 °C

**1.8. αGDH-TPI**

- 34 units required for each plate
- Units per bottle vary from lot-to-lot; calculate volume required for each bottle of enzyme; for example, if the bottle contains 5000 U/4.8 ml, for 34 U use 32 µl (34 × 4.8/5000 = 32)
- Added directly to working reagent, as per instructions, on day of analysis
- Store at +4 °C

**1.9. Working reagent**

- Make up on day of analysis per plate during 15-minute plate incubation
- Add the following to a 50 ml Falcon tube using a pipette:
  - 20 ml Tris buffer
  - 1 ml ribose-5-phosphate at 82.5 mg/ml
  - 1 ml NADH at 3.75 mg/ml
  - 32 µl GDH-TPI (see above; depends on activity of purchased stock)
  - 80 µl 1% Tween-20
- Invert ten times to mix
- Store at +4 °C until use

**2. Procedure**

- 20 samples plus 3 quality control (QC) samples can be analysed in duplicate on a 96-well microplate
- The identification numbers of the samples, QC and batch number should be recorded in a laboratory notebook, together with the mass of reagents as weighed, and incubation timings

**2.1. Preparation of equipment**

- The following steps must be taken to prepare equipment and reagents:

2.1.1. Turn on the incubator and allow it to reach 37 °C (see **note 5**, below)
2.1.2. Turn on the refrigerated centrifuge and set it to cool to 4 °C
2.1.3. Turn on the plate reader and associated computer. Set temperature to 37 °C and allow at least 20 minutes for temperature to equilibrate (see **note 6**, below)
2.1.4. Remove NADH, R5P and TPP from –20 °C storage and allow to equilibrate to room temperature
2.1.5. Remove the controls and samples out from –70 °C storage and allow them to equilibrate to room temperature
2.1.6. Label a corresponding Sarstedt 2 ml tube for each of the samples

**2.2. Preparation of hemolysates**

2.2.1. Once the erythrocyte samples are thawed, mix the samples thoroughly by inversion
2.2.2. To lyse the cells, mix 1 volume of washed erythrocytes with 2 volumes of deionised water (see **note 7**, below). Hemolysates may be stored at –70 °C prior to assay if required
2.2.3. Mix thoroughly and leave at room temperature for 10 minutes for lysis to occur (see **note 8**, below)

**2.3. Analysis**

2.3.1. Centrifuge the samples and controls in the refrigerated centrifuge for 10 minutes at 4000 × *g*

2.3.2. Produce ten-fold dilution of sample and control hemolysates with Tris buffer; e.g. 50 µl plus 450 µl Tris buffer in a 2 ml Sarstedt tube. Invert ten times to mix
2.3.3. Pipette 30 µl quadruplicates of each sample and QC as per the plate map in **Figure P1**. The QC samples should be assayed at the positions shown, ensuring that the position of any individual QC is varied from assay to assay. Assay buffer is used for the four blanks
2.3.4. Add 15 µl Tris to rows A, B, E, F (basal) and 15 µl TPP to rows C, D, G, H (activated) using a multichannel pipette
2.3.5. Cover the plate with a self-adhesive sealer and mix the plate on the plate shaker at 600 RPM for 30 seconds
2.3.6. Carefully place the plate in the incubator at 37 °C for 15 minutes
2.3.7. Prepare working reagent (see step 1.9)
2.3.8. Using a multichannel pipette, add 200 µl of working reagent per well
2.3.9. Mix on the plate shaker at 600 RPM for 30 seconds (do not cover)
2.3.10. Inspect wells for bubbles, if one is present it is advisable to burst it with a clean micropipette tip
2.3.11. Read each well of the plate at 340 nm 60 times at 1 minute intervals, with reader set to briefly shake the plate for 5 seconds before each read

**2.4. Calculation of results**

- The first 40 readings, where the temperature of the plate equilibrates with that of the reader, are not included in the calculations.

2.4.1. Calculate the Δ change in absorbance for each well, during the last 20 readings

The mean blank rate is calculated, and subtracted from every "basal" and "activated" rate. It is advisable to check the four estimates of "blank" and if one deviates significantly from the other three it should be discounted.

In the assay each sample is measured in duplicate with addition of TPP (activated) and in duplicate without addition of TPP (basal). Duplicate measurements are required for each to allow interpretation of reproducibility of the pipetting of samples and reagents. Before the ETKAC can be calculated the imprecision of the activated and basal duplicates needs to be assessed; ETKAC should not be calculated if imprecision of duplicate measurements exceeds 10% for either the basal or activated measurement. To calculate imprecision of duplicates use the following formula:

Δ change in absorbance duplicate 1/Δ change in absorbance duplicate 2

A ratio of 0.9–1.1 is acceptable.

2.4.2. Calculate the mean of each activated and basal duplicate pair

For each sample, divide the mean of the activated duplicate wells by the mean of the basal duplicate wells to calculate the ETKAC.

**2.5. Quality control procedures**

It is recommended that the kinetic trace of the readings are checked for anomalies, such as spikes (which indicates a plate reader malfunction, a bubble or electrical interference) or tapering of the reaction (which indicates insufficiency of reagents).

Samples with basal and/or activated duplicate ratios outside range of 0.9–1.1 should be rerun. If poor duplicate ratios occur regularly, this can be due to either a user error (e.g. poor pipetting technique) or mechanical error (e.g. plate reader malfunction).

*Internal QC material*

It is recommended that 3 separate internal control samples, as well as a reagent blank are run with each batch. The mean ETKAC value and the delta change in absorbance, for both the activated and basal duplicates should be recorded for each level of QC and the reagent blank. The rolling mean, standard deviation and %CV should be calculated for each recorded parameter. After 10 batches have been run the mean ±2 standard deviations of ETKAC values can be used as acceptance limits. The other parameters can be used to aid in interpretation of "failed" batches.

ETKAC assay protocol
NIHR BRC Nutritional Biomarker Laboratory, University of Cambridge
Kerry S. Jones, Damon A. Parkington, Lorna J. Cox, Albert Koulman

To produce internal QC material, washed erythrocytes from single donors should be lysed in bulk following the protocol above for preparation of hemolysates. The hemolysate should then be aliquoted and stored at –70 °C for analysis with each batch. It is important not to mix donations from different donors because clotting is likely to occur, as in a transfusion reaction.
QC 'A': Ideally produced from blood drawn from a thiamine deficient donor. If it is not possible to identify a thiamine deficient donor, a donor with an ETKAC close to the cut-off should be selected.
QC 'B': Should be produced from blood drawn from a donor without thiamine deficiency
QC 'C': "Low Activity" can be derived from any single-donor human washed erythrocyte sample, diluted 1:1 with physiological saline. This is to assess the robustness of the assay in samples with lower concentration of enzyme (for example in basal samples in thiamine deficiency).
-----------------------------------------------------------------------------------------------------------
*Notes for the measurement of ETKAC*
**Note 1.** To achieve acceptable analytical imprecision, it is crucial that the incubation temperature (37 °C) is maintained evenly across the plate during the read stage; the activity of the enzymes used for these reactions is temperature-dependent. The Thermo Multiskan plate reader is specified because of the design of the heating elements; unusually, the chamber is heated by full-sized plates above and below the microplate. Most microplate readers have chambers heated from one corner and cannot be used for this assay. The protocol described in Appendix A can be followed to assess the imprecision of a plate reader specifically for this assay and it is recommended that this protocol is followed before commencing ETKAC analysis and then yearly, or when troubleshooting. The protocol in Appendix A can also be used when assessing other plate readers for suitability with this assay.
**Note 2.** Any pipettes which conform to ISO8655 can be used.
**Note 3.** In the assay, absorbance is read repeatedly down through the well; each time the plate reader lands on one column of wells the position will be slightly different. Therefore, in order to have a constant path length it is necessary to ensure that the meniscus is flat, and this is achieved with the use of Tween 20.
**Note 4.** The use of a Pastette is recommended rather than a pipette due to the viscosity of Tween 20.
**Note 5.** The specifications of the plate reader state that the room temperature must be at least 5 C° below the set temperature and laboratories may require cooling air conditioning to achieve this.
**Note 6.** It is recommended that the plate reader is programmed to run the protocol before commencing analysis.
**Note 7.** For example, 200 µl washed erythrocytes and 400 µl of deionised water. The minimum recommended volume is 20 µl washed erythrocytes and 40 µl of deionised water.
**Note 8.** The hemolysate can be stored at –70 °C post-analysis for repeat batches or other assays.

---

**Table P1. Reagents required for ETKAC assay**

| Reagent | Supplier[b] | Product Code | CAS number | Storage |
|---|---|---|---|---|
| Trizma base (for Tris buffer) | Sigma brand | T6791 | 77-86-1 | Room temperature |
| Thiamine pyrophosphate (TPP)[a] | Sigma | C8754-5G | 154-87-0 | –20 °C |
| Ribose-5-phosphate | Sigma | R7750-1G | 18265-46-8 | –20 °C |
| β-Nicotinamide adenine dinucleotide, reduced disodium salt hydrate (NADH) | Sigma | N8129-1G | 606-68-8 | –20 °C |

| | | | | |
|---|---|---|---|---|
| α-Glycerophosphate Dehydrogenase-Triosephosphate Isomerase from rabbit muscle (αGDH-TPI) | Sigma | G1881-5KU | − | 2–8 °C |
| 37% Hydrochloric Acid | Sigma | H1758 | 7647-01-0 | Room temperature |
| Tween 20 | Sigma | | | Room temperature |

[a]Also referred to as thiamine diphosphate (ThDP)
[b]All chemicals can be sourced through Sigma brand supplier: Merck Life Science UK Ltd, Gillingham, Dorset, UK

**Figure P1. Plate map for ETKAC analysis.** Sample and QC (30 µl) are added to each of four wells on the plate. To the shaded rows A, B, E, F (basal wells) is added 15 µl Tris buffer and to rows C, D, G, H (activated wells) 15 µl TPP is added. Abbreviations: BLK, reagent blank (Tris buffer); QC, internal quality control sample; 1–20, samples.

| Row/ Column | 1 | 2 | 3 | 4 | 5 | 6 | 7 | 8 | 9 | 10 | 11 | 12 |
|---|---|---|---|---|---|---|---|---|---|---|---|---|
| A | BLK | 1 | 3 | 5 | 7 | 9 | QC | 12 | 14 | 16 | 18 | 20 |
| B | BLK | 1 | 3 | 5 | 7 | 9 | QC | 12 | 14 | 16 | 18 | 20 |
| C | BLK | 1 | 3 | 5 | 7 | 9 | QC | 12 | 14 | 16 | 18 | 20 |
| D | BLK | 1 | 3 | 5 | 7 | 9 | QC | 12 | 14 | 16 | 18 | 20 |
| E | QC | 2 | 4 | 6 | 8 | 10 | 11 | 13 | 15 | 17 | 19 | QC |
| F | QC | 2 | 4 | 6 | 8 | 10 | 11 | 13 | 15 | 17 | 19 | QC |
| G | QC | 2 | 4 | 6 | 8 | 10 | 11 | 13 | 15 | 17 | 19 | QC |
| H | QC | 2 | 4 | 6 | 8 | 10 | 11 | 13 | 15 | 17 | 19 | QC |

---

**Appendix A – Protocol to assess the suitability of plate reader performance for the ETKAC assay**
The purpose of this procedure is to determine the level of within-plate imprecision due solely to the variation of temperature within the incubation chamber of the plate reader. It is not possible to measure this temperature directly in each well; the plate reader recorded "37 °C" applies only to a single site within the chamber. In this procedure the normal protocol is followed except only one sample is used and processed in bulk before transferring to 96-well plate for reading. This means that any variation in rate between wells results only from temperature differences between the wells.

ETKAC assay protocol
NIHR BRC Nutritional Biomarker Laboratory, University of Cambridge
Kerry S. Jones, Damon A. Parkington, Lorna J. Cox, Albert Koulman

It is recommended that this procedure is run at least once a year and after any engineer service visit, as the assay will require a plate reader to exceed the manufacturer specifications. It may

also be useful to follow this procedure when troubleshooting. Routine inspections and checks of the plate reader by an approved service engineer are also recommended.

**A1. Procedure**

1. Turn on the incubator and allow it to reach 37 °C
2. Turn on the plate reader and associated computer. Set temperature to 37 °C and allow at least 20 minutes for temperature to equilibrate
3. Remove sufficient vials of a QC material to provide 300 µl hemolysate and allow to equilibrate to room temperature on a roller mixer
4. Mix thoroughly by inversion. Centrifuge the samples and controls in the refrigerated centrifuge for 10 minutes at 4000 × *g*
5. Pipette 300 µl QC material hemolysate and 2.7 ml Tris buffer into a conical flask or Falcon tube
6. Add 1.5 ml TPP (to ensure higher rates) and swirl gently to mix
7. Incubate for 15 minutes at 37 °C
8. Prepare working reagent (see step 1.9 of "Protocol for the measurement of erythrocyte transketolase activity coefficient (ETKAC)")
9. Add 19.2 ml working reagent and swirl gently to mix
10. Pipette 200 µl of the mixture into each well of the 96-well plate
11. Mix on the plate shaker at 600 RPM for 30 seconds (do not cover)
12. Inspect wells for bubbles, if one is present it is advisable to burst it with a clean micropipette tip
13. Read each well of the plate at 340 nm 60 times at 1-minute intervals, with reader set to briefly shake the plate for 5 seconds before each read

**A2. Calculation of results**

1. The first 40 readings, where the temperature of the plate is being brought to that of the reader, are not included in the calculations
2. Calculate the Δ change in absorbance at 340 nm for the last 20 readings for each well of the plate
3. Inspect traces for "spikes" if any individual rate is aberrant. Wells in which the rate has been affected by a spike or non-linearity or from mis-pipetting should be excluded from the calculations
4. Calculate the mean, standard deviation and %CV of the Δ change in absorbance measurements for each row, each column and for the whole plate
5. Inspect the results to look for trends in rate (i.e. in temperature) and also to look for edge effects (centre warmer than edges)
6. If the CVs of the rows or columns demonstrate unevenness in the enzyme rate and thus in the temperature in the wells, or if there is an "edge effect", the part of the plate affected is not suitable for this assay. The recommendation is that for rows and columns with the plate the CV should be < 2% and for the plate overall, < 3%
7. Using notional "blank" rates (e.g. average blank rates obtained from QC data), calculate "ETKAC" for each set of four wells in the same way as would be done for an assay. Ideally each calculated ETKAC will be 1.00 because the same reaction is occurring in every well. Inspect the results for deviations from 1.00; this provides an indication of the within-batch imprecision to be expected from an assay
